# Dr. Graves on the Diagnosis and Treatment of Certain Affections of the Heart—Pericarditis

**Published:** 1842-06-04

**Authors:** 


### BIBLIOGRAPHICAL NOTICE.

*On the Diagnosis and Treatment of certain affections of the Heart—Pericarditis.* By ROBERT J. GRAVES, M. D., &c. &c.

Dr. Graves has published a paper in the Dublin Journal for the last month (May) upon certain affections of the heart. The first part of it relates to pericarditis, of which we have given two cases lately observed in the Philadelphia Hospital. The disease when treated at a tolerably early stage is seldom fatal; the cases which terminate unfavourably are almost always those which from the first are neglected, or which are complicated with chronic diseases of long standing, or severe inflammation of other organs.

In the first case given by Dr. Graves, the disease began with rheumatism two months previously; this was followed by dyspnœa and anasarca.

"On admission, his surface was cold, lips and hands livid, feet swollen, and belly distended. He suffered from dyspnœa; cough, with bloody expectoration; his eyes were staring and protruded; face tumid; jugulars turgid, but not pulsating; pulse 70, regular, but small and weak; respiration 28; urine scanty and highly albuminous; extreme debility. The left lobe of the liver occupied the epigastric region, in which situation alone pressure caused pain. He had slight pain in the right shoulder. There was no dulness except over the lower and back part of both lungs, where the respiration was weak and accompanied by a moist crepitus; the cardiac region sounded duller than natural. The motions of the heart were evident, strong, diffused, and were accompanied, not by the two natural sounds, whose duration and tone are so different from each other, but by two loud, prolonged sounds, of equal duration but of different tones; the first was a *bruit de scie*, the second was a musical sound, closely resembling the noise made by rubbing the moistened finger on glass. These phenomena were only heard at the base, and were quite inaudible at the apex of the heart; but they extended from the base along the aorta, and were very distinct under both clavicles, particularly the left; they were not heard either in the carotids or in the cervical portions of the subclavians. In no situation was there the least frémissement; no thrill in any of the arteries of the neck or upper extremities; no abnormal sound over the abdominal aorta. The next day, his condition was much the same, except that instead of the musical sound we had a well-marked and loud *leather creak*, very much prolonged and masking the normal second sound, and a strong frémissement was felt over the base of the heart; there was no increase of dulness. The pulse continued regular, 72; the respiration only 20; but he was evidently sinking, and on the following morning he died.

"*Post Mortem.*—General anasarca; both pleural cavities were occupied by a large quantity of fluid, upon which the lungs floated; on the left side the heart was bedded in the lung, and both were carried into close apposition with the anterior parietes of the chest, so as to bring the heart into extensive contact with the sternum and costal cartilages. There was no fluid in the pericardium, but its surfaces were coated with lymph, shreds of which extended from one surface to the other at the base of the heart. In this situation, the lymph appeared to have been very recently effused; it was easily removed, and presented an irregular honeycomb appearance. At the apex of the heart, the opposed membranes were firmly united. The heart itself was hypertrophied and both its ventricles dilated. All the valves, the endocardium, the aorta and pulmonary artery were quite free from the least trace of disease."

Dr. Graves remarks that the general symptoms were obscure, but the physical signs were even more liable to mislead, because they rather indicated disorder of the valves than of the pericardium, until the decided creaking sound rendered the case clear. The case, however, is by no means unique; it is not very uncommon to find that a double, rough, or even a rasping sound is heard when the pericardium is apparently the only part of the heart which is inflamed. Dr. Graves thinks that the sounds heard in pericarditis may always be distinguished by the greater extent in which they are heard, and by their appearing to the ear to proceed from a more superficial part of the heart than those dependant upon endocarditis or other affections of the internal membrane. We believe that in many cases this difference in the extent and in the seat of the sounds is obvious enough, but there are other cases in which the abnormal sounds certainly depend in part upon the morbid muscular contraction, and although they are caused by pericarditis, they are not always formed in the pericardium itself.

The next case is very curious; the rheumatic inflammation attacked the heart before the joints. Dr. Graves remarks that physicians have been hitherto too prone to attribute attacks of pericarditis, occurring in connexion with rheumatism, to metastasis. This opinion was certainly at one time current, but it is now well understood that attacks of pericarditis generally arise during the height of rheumatism, when the pain and inflammation of the joints are most intense.

"A woman, æt. 19, named Fitzgerald, was admitted September 1st, 1841, into the hospital, labouring under febrile symptoms of a trifling character. She complained principally of headach, with loss of sleep. Her pulse was quick and her tongue foul. For these symptoms she was treated, and every thing seemed going on favourably, till September 5th, when the following observation was made:

"Face pallid and anxious; breathing hurried, 40; alæ nasi dilated at each inspiration; pulse has fallen from 90 to 50, very *weak*, *irregular and intermittent;* no cough or pain in the chest; no palpitation; physical examination did not detect disease any where, except over the cardiac region, in which there was a distinct rubbing sound, accompanying both sounds of the heart. It was most intense at the apex of the organ, and appeared to accompany the first sound more particularly. It was attended with a very perceptible frémissement; in no situation had it the character of a "*soufflet.*" The impulse of the heart was exceedingly strong, and its sounds very loud. She was cupped over the heart and put on the use of calomel and opium, five grains of the former with one of the latter, every fourth hour.

"September 6th. Countenance much improved; pulse 72, full and soft, but still irregular and intermittent; respiration 28; alæ nasi not dilated; no pain in any part. The *friction* sound is still very evident, though less intense, particularly at apex of heart; no dulness, impulse stronger than on yesterday, sounds of heart very distinct. Blister over the heart, and continue the pills of calomel and opium.

"7th. Mouth sore; pulse 76, small, soft, regular, *without any intermission;* respiration 28, countenance good; the impulse and sounds of heart are both good; the friction is barely audible, being most intense over the right side of heart. Cont. pills.

"8th. No trace of frottement; the sounds and impulse of heart natural; pulse 80, regular and soft.

"10th. Was last night attacked with pains in the loins, knees, shoulders, wrists, and ankles. These joints are now exceedingly painful, red and swollen. Pulse 80, small and soft."

"In some cases of pericarditis the heart's action becomes increased in strength for many hours before any physical sign of pericarditis can be detected, and before the pain is felt in the region of the heart." This is perfectly correct; the first attack of inflammation excites the muscular contraction of the heart; it depresses at a later period when effusion of lymph or serum has taken place. The pulse may fall in pericarditis, or it may remain natural; in itself it is therefore of little or no value as a diagnostic sign.

The third case is the common one of pericarditis occurring during the height of the rheumatic inflammation; which generally is a very manageable variety of the disease, and terminated, as is usual, favourably.

The last case is one of pericarditis accompanied with a peculiar eruption over the body; evidently an accidental complication. The friction sound was more limited, because the heart was not enlarged as in the first case.

